# Contrast-Induced Encephalopathy: A Clinical Conundrum

**DOI:** 10.7759/cureus.31360

**Published:** 2022-11-11

**Authors:** Arunava Saha, Shari Mitra

**Affiliations:** 1 Internal Medicine, Saint Vincent Hospital, Worcester, USA; 2 Ear, Nose, and Throat (ENT), Government Medical College and Hospital, Surat, IND

**Keywords:** contrast toxicity, adverse event, altered sensorium, metabolic encephalopathy, iv contrast

## Abstract

Contrast-induced encephalopathy (CIE) is a rare but well-known complication of mostly intra-arterial contrast administration and presents with a variety of neurological deficits due to disruption of the blood-brain barrier. We present a case of CIE after administration of intravenous contrast for computed tomography pulmonary angiogram (CTPE).

A woman in her mid-70s with history of chronic obstructive pulmonary disease (COPD) presented with progressively worsening shortness of breath. She was diagnosed with multifocal pneumonia and started on IV antibiotics, IV steroids, and bilevel positive airway pressure (BiPAP) ventilation. A CTPE was done to rule out a pulmonary embolism during which she received 100 cc of Isovue 370 (iopamidol 76%), a low-osmolar, non-ionic, monomeric, iodine-based contrast agent. Within minutes of the contrast administration, the patient developed confusion and agitation with elevated blood pressure. Neurological evaluation revealed no gross focal motor or cranial nerve deficits and bilateral 2+ reflexes with mute plantar reflexes. Laboratory investigations were unchanged. She was shifted to the ICU but continued to remain drowsy and disoriented. CT brain done within two hours of onset revealed no intracranial abnormality. She was managed conservatively with IV fluids, neuro-checks, and blood pressure control. Her sensorium improved within 48 hours with supportive treatment. Repeat neuroimaging was not performed. She was discharged after four days with the resolution of her respiratory symptoms.

CIE is a known but uncommon complication associated with the use of intraarterial contrast media but has been found to occur even after intravenous administration, which has been reported only once in literature. The presentation is highly variable, ranging from headache to coma, with transient cortical blindness being the most commonly identified. The diagnosis requires a high index of suspicion, and brain imaging is usually pathognomonic; however, cases in the absence of radiological signs have also been diagnosed. Typically, symptoms resolve within 48-72 hours and the disease runs a benign course, but cases of persistent neurological deficit and even cases of fatal cerebral edema have been reported.Treatment is usually supportive with intravenous hydra­tion and anticonvulsants and the occasional use of IV steroids and mannitol with favorable outcomes.

## Introduction

Contrast-induced encephalopathy (CIE) is a rare but well-known complication of percutaneous carotid and coronary interventions and presents with a variety of neurological deficits that are attributed to the disruption of the blood-brain barrier [1-3]. All forms of contrast media, which include ionic, non-ionic, low osmolarity, iso-osmolar, and high osmolarity solutions, have been reported to cause CIE [1,2]. The most common manifestations include encephalopathy, seizures, motor and sensory disturbances, transient cortical blindness, and focal neurological deficits. Most of the symptoms usually resolve within 48-72 hours, but there have been cases of fatal cerebral edema and death secondary to CIE associated with the use of ionic high-osmolar contrast agents [4]. Even though the exact etiology remains unknown, a few risk factors identified over time include chronic hypertension, diabetes mellitus, renal impairment, administration of large volumes of iodinated contrast, percutaneous coronary intervention, impaired cerebral autoregulation, and previous adverse reactions to iodinated contrast [5]. Almost all but one case reported in the literature have occurred post the intra-arterial administration of contrast media [6]. Diagnosis is confirmed by typical radiological signs on a non-contrast CT scan of the brain in a symptomatic patient after excluding thromboembolic and hemorrhagic complications; however, cases have been known to present without the typical radiological signs [7]. CIE developed after intravenous contrast administration for a CT pulmonary embolism study in our patient, and the brain imaging was found to be normal.

## Case presentation

A woman in her mid-70s with a history of diabetes mellitus, hypertension, Hashimoto’s thyroiditis status post partial thyroidectomy, and chronic obstructive pulmonary disease (COPD) presented with complaints of progressively worsening shortness of breath and chest tightness for one week, which worsened over the course of one day. On presentation to the emergency department, she was found to be tachycardic to 140s in atrial fibrillation and hypoxic to the 70s. Respiratory examination revealed diffuse wheeze. She was started on a non-rebreather and managed with a dose of IV Metoprolol. Laboratory investigations revealed leukocytosis with bandemia, negative cardiac enzymes, and respiratory acidosis with CO_2_ retention on the arterial blood gas (ABG). Chest X-ray demonstrated features of multifocal pneumonia. She was started on antibiotics in the form of ceftriaxone and azithromycin, IV methylprednisone, and IV fluids. She was also started on bilevel positive airway pressure (BiPAP) ventilation for increased work of breathing and increasing oxygen requirements. A computed tomography pulmonary angiogram (CTPE) was planned to rule out a pulmonary embolism in the setting of new-onset atrial fibrillation and worsening hypoxia. A total of 100 cc of Isovue 370 (iopamidol 76%), a low-osmolar, non-ionic, monomeric, iodine-based contrast agent, was administered during the procedure. Within minutes of the contrast administration, the patient developed an altered sensorium in the form of confusion and agitation. She was also found to have elevated blood pressure in the 180/120 mmHg range. She was shifted to the ICU but continued to remain drowsy and disoriented. The CTPE was found to be negative. Neurological evaluation revealed no gross focal motor or cranial nerve deficits and bilateral 2+ reflexes with mute plantar response. There was no neck stiffness or any seizure-like activity. Stat laboratory investigations, including a complete blood count and comprehensive metabolic panel, were unchanged. Thyroid-stimulating hormone (TSH) and thyroid profile were within normal limits. ABG did not show any hypercapnia. Blood cultures were negative with normal procalcitonin, hence infectious etiology was ruled out and antibiotics were deferred. A CT brain done within two hours of symptom onset revealed no intracranial abnormality and only chronic microangiopathic senescent changes. She was started on hourly neuro-checks, a nicardipine drip to control her blood pressure, which was tapered and stopped within six hours, and IV fluids to promote excretion of the contrast agent. She was also continued on the instituted treatment for her pneumonia including steroids. Within 48 hours of supportive management, her sensorium improved. A repeat neuroimaging in the form of MRI brain was planned, but her symptoms improved prior to it and she requested to defer it in the setting of her claustrophobia. Apixaban was added for new-onset atrial fibrillation, and she was discharged after four days without any neurological deficits with the resolution of her respiratory symptoms.

## Discussion

CIE is a known but uncommon complication associated with the use of intra-arterial contrast media during percutaneous carotid and coronary interventions, with an incidence ranging between 0.3% and 1.0%, but higher rates have been documented with the use of hyperosmolar iodinated contrast agents [4]. In this report, we presented a highly probable case of CIE following intravenous administration of a small amount of non-ionic, low-osmolar contrast agent, with severe symptoms and spontaneous recovery.

Although there have been more than 50 cases of CIE identified in the literature since the clinical description in 1970, all but one have occurred following angiographic procedures and intra-arterial administration of contrast [5,6]. This supports the hypothesis that the symptoms are a result of the chemotoxic effect of hyperosmolar media resulting in microvascular sludging and arterial spasms, causing shrinkage of the endothelial cells and disruption of tight junctions. The accumulation of iodinated contrast has also been known to cause a localized neurotoxic effect on the blood-brain barrier by increasing the expression of endothelin, amplifying endothelial cell permeability, and resulting in cerebral edema [7-9]. However, in our case, the contrast was administered intravenously for a CTPE study. The patient did not have any renal impairment and had no prior history of neurological interventions but did have diabetes and systemic hypertension.

The presentation for CIE has been found to be highly variable, ranging from subtle symptoms, such as headache, to more extreme features such as coma or unresponsiveness. Transient cortical blindness is the most common manifestation [10]. The literature review indicated that symptoms of neurological dysfunction presented within minutes to hours after contrast agent administration, and most patients fully recovered within 48-72 hours with supportive management [7-9]. In our patient, the symptoms resolved completely within 48 hours, which was consistent with the clinical course of CIE. We suspect that the severity of symptoms in our patient was related to her advanced age, hypertension, and other risk factors, which may have increased blood-brain barrier permeability, resulting in neurotoxicity and CIE.

A small dose of contrast media, even as low as 25 ml, has been reported to cause CIE and can occur with both high- and low-osmolar contrast agents. There has been no defined amount of dye beyond which CIE is certain or more likely to occur [5]. The culprit in our case was Iopaque 370, a low-osmolar, non-ionic, monomeric, iodine-based contrast agent that contains 755 mL of iopamidol equivalent to 370 mg of iodine per milliliter. As 100 mL was used in our patient, the diagnosis of CIE was highly presumable even without the associated typical radiological signs.

The diagnosis of CIE requires a high index of suspicion, and brain imaging is usually pathognomonic showing contrast enhancement in the cortical, subarachnoid, striatal spaces, or cerebral edema, which are important for the differentiation of CIE from other pathologies such as thromboembolism and hemorrhage [8,9,11]. Typically, symptoms resolve within days and the disease runs a benign course, but cases of persistent neurological deficit and even fatal cerebral edema secondary to contrast injection have been reported. However, the literature review revealed multiple cases where patients were diagnosed with CIE in the absence of the classic radiological signs, which was the case with our patient [7]. Cerebrospinal fluid (CSF) examination has also been found useful to rule out subarachnoid hemorrhage, and high concentrations of iodine contrast in the CSF and serum support contrast extravasation [12]. The differentials considered for our patient included hypoxia and hypercapnia (ABG was normal), hypoglycemia (blood glucose levels were normal), transient ischemic attack (altered sensorium took more than 24 hours to resolve), cerebrovascular accident and venous sinus thrombosis (CT brain without contrast was normal, there were no focal neurological deficits), toxic metabolic encephalopathy (electrolytes and urea were normal), meningitis (no neck stiffness and symptoms resolved without antibiotics), status epilepticus (no seizure-like activity), and thyrotoxicosis (TSH and thyroid profile were normal).

The mainstay of management is supportive treatment with intravenous hydra­tion and anticonvulsants for the control of sei­zures. IV steroids and mannitol have been used sporadically with favorable outcomes. In patients with renal insuf­ficiency on dialysis who develop CIE, hemodialysis has proven beneficial [5,8,9,11]. Our patient was managed with hydration and steroids, which she was receiving for her underlying COPD exacerbation, and her symptoms resolved.

As a limitation of our case report, a brain MRI was not performed despite a negative CT brain. MRI brain has been found to frequently reveal the presence of vasogenic edema with gyral swelling and increased signal on T2-weighted and fluid-attenuated inversion recovery (FLAIR) sequences, with increased intensity on diffusion-weighted imaging (DWI) sequences. Also, a repeat CT brain was not performed due to the complete resolution of symptoms within 48 hours of onset.

## Conclusions

Our case highlights a case of CIE that is usually attributed to intra-arterial administration of contrast but can present even with intravenous injection, which clinicians need to be aware of.

The presentation of CIE is widely variable, ranging from cortical blindness to encephalopathy, seizures, and hemiparesis. In any acutely confused patient with a history of recent contrast administration, it is important to evaluate and carry out relevant investigations, as CIE is often a diagnosis of exclusion. Although there is no specific treatment for CIE, the management is usually supportive with an excellent prognosis.
